# Elevated expression of ZNF217 promotes prostate cancer growth by restraining ferroportin-conducted iron egress

**DOI:** 10.18632/oncotarget.12753

**Published:** 2016-10-19

**Authors:** Xingkang Jiang, Changwen Zhang, Shiyong Qi, Shanqi Guo, Yue Chen, E Du, Hongtuan Zhang, Xiaoming Wang, Ranlu Liu, Baomin Qiao, Kuo Yang, Zhihong Zhang, Yong Xu

**Affiliations:** ^1^ Department of Urology, The Second Hospital of Tianjin Medical University, Tianjin Institute of Urology, Tianjin 300211, China; ^2^ Department of Oncology, First Teaching Hospital of Tianjin University of Traditional Chinese Medicine, Tianjin 300112, China

**Keywords:** ferroportin, prostate cancer, MAZ, EZH2, ZNF217, **Abbreviations:** Prostate cancer (PCa), Zinc-figure protein 217 (ZNF217), reverse transcription and quantitative real-time PCR analysis (qRT-PCR), Chromatin imunoprecipitation (ChIP), Ferroportin (FPN)

## Abstract

Although we and other studies indicated ZNF217 expression was increased in prostate cancer (PCa), the factors mediating its misregulated expression and their oncogenic activity remain largely unexplored. Recent evidence demonstrated that ferroportin (FPN) reduction lead to decreased iron export and increased intercellular iron that consequently aggravates the oncogenic effects of iron. In the present study, ZNF217 was identified as a transcriptional repressor that inhibits FPN expression. Increased of ZNF217 expression led to decreased FPN concentration, coupled with resultant intracellular iron retention, increased iron-related cellular activities and enhanced tumor cell growth. In contrast, decreased of ZNF217 expression restrained tumor cell growth by promoting FPN-driven iron egress. Mechanistic investigation manifested that ZNF217 facilitated the H3K27me3 levels of FPN promoter by interacting with EZH2. Besides, we also found that MAZ increased the transcription level of ZNF217, and subsequently inhibited the FPN expression and their iron–related activities. Strikingly, the expression of MAZ, EZH2 and ZNF217 were concurrently upregulated in PCa, leading to decreased expression of FPN, which induce disordered iron metabolism. Collectively, this study underscored that elevated expression of ZNF217 promotes prostate cancer growth by restraining FPN-conducted iron egress.

## INTRODUCTION

Zinc-figure protein 217 (ZNF217), a member of the Kruppel-like family of transcriptional factors, was firstly amplified in breast cancer and associated with aggressive tumor behavior and poor clinical prognosis [1, 2]. ZNF217 encodes eight predicted C_2_H_2_ zinc finger motifs and a proline-rich transactivation domain at the C-terminus [3–5]. In addition to regulating breast cancer progression, later studies also identified that ZNF217 plays a critical role in other cancers, such as ovarian cancer, esophagus cancer and prostate cancer [3, 6–10]. Until now, the intrinsic regulatory mechanisms of ZNF217 exerts its oncogenic activity are still elusive, and only a few targets have been verified in tumor cells. For example, biochemical purification studies identified ZNF217 as a constituent of several related transcriptional co-repressor complexes, which contains the histone deacetylases and histone methyltransferases [11]. Besides, ZNF217 has also been identified as a co-repressor of the C-terminal binding protein (CTBP) complex [12–14] and ERalpha [15]. However, other studies also suggested that ZNF217 could positively regulates the gene expression of specific target genes [16, 17]. Therefore, further efforts are still required to clarify the actual role of ZNF217 in different settings that will be valuable to better understand of the molecular bases of malignancy and to better design of target therapeutics.

Ferroportin (FPN), a multiple-transmembrane domain protein transmembrane protein, is the only known iron exporter in mammalian cells. FPN is expressed in many iron-exporting cells, including placental syncytiotrophoblasts, duodenal enterocytes, hepatocytes, reticuloendothelial macrophages and even cancer cells [18–20]. Reduced expression of FPN on the cell surface lead to an increase in intracellular iron content and decreased iron efflux, which consequently makes the tumor cells more aggressive [19, 21, 22]. Hepcidin, the physiologic ligand of FPN, inhibits iron export by inducing internalization, ubiquitination and degradation of FPN protein [22–25]. Other than this, other studies also indicated that FPN expression can also be transcriptionally regulated [26, 27]. Besides, hypermethylation of the FPN promoter was also involved in decreased FPN concentration in tumor cells [26]. However, we still have limited knowledge of decreased FPN expression in cancer cells.

In the present study, we demonstrated that ZNF217 functions as a transcriptional repressor that associates with EZH2 to facilitate H3K27me3 levels of FPN to suppress FPN expression. In addition, elevated myc-associated zinc finger protein (MAZ) levels transcriptionally promotes ZNF217 expression in tumor cells. Therefore, our results define a novel pathway that ZNF217 exerts its oncogenic activity in PCa by restraining FPN-conducted iron egress.

## RESULT

### Elevated ZNF217 expressionaggravates iron-related tumor cell growth

**Figure 1:**
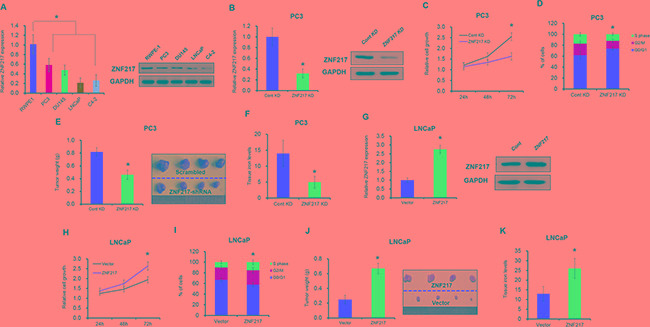
Elevated ZNF217 expression aggravates iron-related tumor cell growth **A.** The mRNA and protein level of ZNF217 in four different PCa cell lines (i.e. PC3, DU145, LNCaP and C4-2) and prostatic epithelial cell line RWPE-1 by qPCR and Western blot analysis, respectively. **B, F.** The mRNA and protein level of ZNF217 were assessed upon ZNF217 knockdown in PC3 cells or overexpression in LNCaP cells. **C, G.** The cell growth were examined upon ZNF217 knockdown in PC3 cells or overexpression in LNCaP cells. **D, H.** The cell cycle was detected upon ZNF217 knockdown in PC3 cells or overexpression in LNCaP cells. **E, J.** The final tumor weight of tumors derived from ZNF217 knockdown PC3 cells or ZNF217 transfected LNCaP cells. **F, K.** The tissue iron levels in tumor samples upon ZNF217 knockdown in PC3 cells or overexpression in LNCaP cells. Data depict the mean ± SD and representative of six independent experiments. Asterisk (^*^) indicates P < 0.05

Although we and other studies have identified the effect of ZNF217 on tumor behaviors, the mechanisms underlying abnormal ZNF217 in iron-related tumors growth remain unexplored. As shown in Figure 1A, the mRNA and protein levels of ZNF217 were significantly upregulated in four different PCa cell lines (i.e. PC3, DU145, LNCaP and C4-2), when compared with prostatic epithelial cell line RWPE-1 (P<0.05). To investigate the biological behavior of ZNF217 on iron homeostasis, two PCa cell lines PC3 and LNCaP were selected to established cell lines with knockdown or overexpression of ZNF217. As illustrated in Figure 1B, ZNF217 knockdown largely inhibited the mRNA and protein levels of ZNF217 in PC3 cells by almost 70% and 60%, respectively (P<0.05). In addition, the growth of PC3 cells was significantly suppressed upon ZNF217 knockdown, as the Alarmar Blue assay showed a 50% decrease in cell growth of PC3 cells at 72 h (Figure 1C, P<0.05). Meanwhile, flow cytometer analysis also revealed a significantly increase of cells in the G0/G1 phase and a decrease of cells in the G2/M phase upon ZNF217 knockdown (Figure 1D, P<0.05). Consistent to *in vitro* results, tumor growth and the final mean volume of the xenograft tumors (6/10) were substantially inhibited almost 50% in ZNF217 knockdown group, when compared with those in control group (8/10) (Figure 1E, P<0.05). Interestingly, we found that tissue iron content in tumor samples was significantly decreased in ZNF217 knockdown group, compared to those of control group (Figure 1F, P<0.05). In contrast, we also transfected ZNF217 overexpression plasmids in LNCaP cells (Figure 1G, P<0.05). Cell proliferation was greatly increased and cell cycle was also enhanced in ZNF217-transfected LNCaP cells (Figure 1H–1I, P<0.05). Besides, we found that overexpression of ZNF217 significantly promoted tumor growth, with the mean weight of xenograft tumors increased almost 2.6-fold in ZNF217 overexpression group (8/10) than control group (6/10) (Figure 1J, P<0.05). Moreover, tissue iron concentration in tumor samples was obviously elevated in ZNF217 overexpressed group (Figure 1K, P<0.05). These combined data demonstrated that aberrant ZNF217 expression modulates iron-related tumor cell growth.

### Increased ZNF217 expression promotes tumor cell iron metabolism

**Figure 2:**
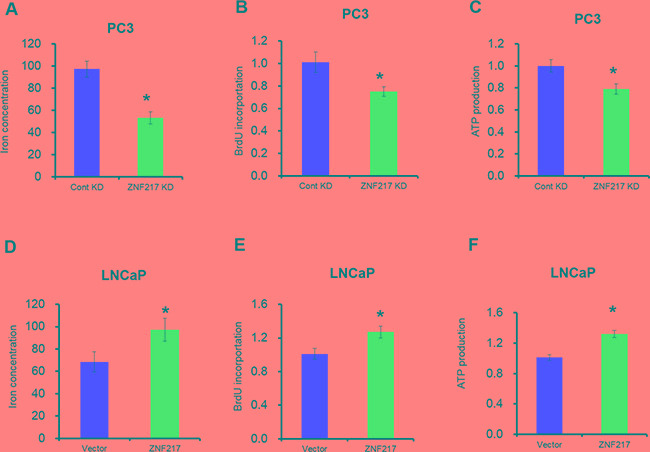
Increased ZNF217 expression promotes tumor cell iron metabolism **A, D.** Intracellular iron content was examined after ZNF217 knockdown in PC3 cells or overexpression in LNCaP cells. **B, E.** DNA replication was assessed upon ZNF217 downregulation in PC3 cells or forced ZNF217 expression in LNCaP cells. **C, F.** Relative ATP levels was determined after ZNF217 knockdown in PC3 cells or ZNF217 overexpression in LNCaP cells. Data depict the mean ± SD and representative of six independent experiments. Asterisk (^*^) indicates P < 0.05

Iron is an essential element for cell growth, as it is involved in DNA synthesis, energy metabolisms and other important cellular processes [18]. To evaluate whether ZNF217 regulates iron metabolisms in tumor cells, the intracellular iron content was measured with fluorescent metallosensor calcein. As illustrated in Figure 2A, ZNF217 knockdown in PC3 cells had lower iron concentration compared with their counterparts (P<0.05). Moreover, we also examined iron-related cellular activities including DNA replication and ATP production by BrdU incorporation assay and ATP assay, respectively. Upon ZNF217 knockdown, DNA replication and ATP production in PC3 were reduced by 23% and 25%, as compared with their scramble controls (Figure 2B–2C, P<0.05). In contrast, ZNF217 overexpression in LNCaP cells increased intracellular iron level compared to controls (Figure 2D, P<0.05). Elevated DNA replication and ATP content were also found in ZNF217 overexpressed LNCaP cells (Figure 2E, P<0.05). Therefore, these above results suggested that increased ZNF217 expression promotes tumor cell iron metabolism.

### ZNF217 inhibits the expression of FPN to modulating iron metabolism

**Figure 3:**
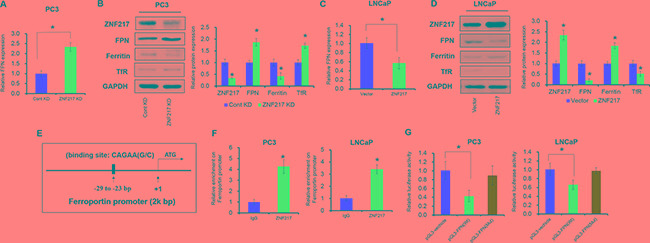
ZNF217 inhibits the expression of FPN to modulating iron metabolism **A, C.** The mRNA level of ZNF217 was detected upon ZNF217 knockdown in PC3 cells or overexpression in LNCaP cells. **B, D.** The protein levels of ZNF217, FPN, ferritin and TfR were assessed upon ZNF217 downregulation in PC3 cells or forced ZNF217 expression in LNCaP cells. **E.** The putative ZNF217 binding site in FPN promoter regions. **F.** ChIP assay showed that ZNF217 expression was increased in the promoter of FPN in PC3 and LNCaP cells upon ZNF217 overexpression. **G.** Luciferase assay showed that ZNF217 binds to the promoter of FPN. Data depict the mean ± SD and representative of six independent experiments. Asterisk (^*^) indicates P < 0.05

To elucidate the underlying mechanism by which ZNF217 exerts its function in iron-related cancer growth, we assessed the FPN expression upon ZNF217 downregulation or upregulation. As illustrated in Figure 3A-3B, FPN concentration was elevated by approximately 2.3-fold and 1.7-fold at mRNA and protein levels in PC3 cells upon ZNF217 knockdown (P<0.05). Consequently, we also found that ZNF217 knockdown decreased intracellular iron levels, as evidenced by the decreased intracellular iron storage protein levels (i.e. ferritin) and increased iron import protein expression (i.e. transferrin receptor 1) (Figure 3B, P<0.05). As shown in Figure 3C-3D, FPN concentration was increased by almost 55% and 45% at mRNA and protein levels in LNCaP cells upon ZNF217 overexpression plasmid (P<0.05). Because of FPN decrease, the intracellular iron levels surged, as reflected by the increased ferritin content and the decreased transferrin receptor 1 levels (Figure 3C–3D, P<0.05). Based on the bioinformatics analysis, we found a putative ZNF217 binding site at -29 to -23 bp within the promoter region of FPN (Figure 3E). To determine whether ZNF217 are bound to the FPN promoter, we performed ChIP assay with the antibody against human ZNF217 in PC3 cells. As showed in Figure 3F, significant enrichment was found within the promoter of FPN upon ZNF217 antibody precipitation, indicating the binding ZNF217 to the promoter of FPN (P<0.05). Besides, ZNF217 overexpression significantly suppressed the relative luciferase activity of pGL3-FPN(Wt) which contains ZNF217 binding sites in FPN promoter regions the by 60%, compared to those of controls (Figure 3G, P<0.05). However, this suppression was not observed for pGL3-FPN(Mut), which contains the deletion of ZNF217 binding site within the FPN promoter (Figure 3G, P<0.05). These data collectively demonstrated that ZNF217 regulates FPN expression to modulating iron metabolism.

### ZNF217 facilities the H3K27me3 levels of FPN promoter by cooperating with EZH2

**Figure 4:**
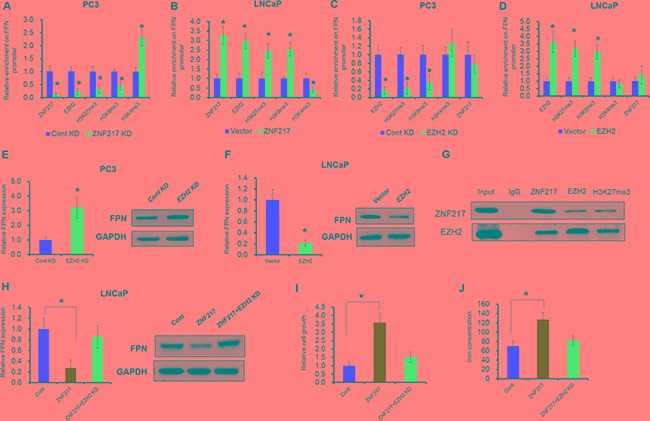
ZNF217 facilities the H3K27me3 levels of FPN promoter by cooperating with EZH2 **A, B.** The binding ability ZNF217, EZH2, H3K27me3, H4K9me3 and H3K4me3 levels on the promoter of FPN were assessed ZNF217 downregulation in PC3 cells or forced ZNF217 expression in LNCaP cells. **C, D.** The binding ability ZNF217, EZH2, H3K27me3, H4K9me3 and H3K4me3 levels on the promoter of FPN were assessed EZH2 downregulation in PC3 cells or forced EZH2 expression in LNCaP cells. **E, F.** The mRNA and protein levels of FPN were examined upon EZH2 knockdown in PC3 cells or overexpression in LNCaP cells. **G.** Co-IP assay showed a directly interaction between ZNF217, EZH2 and H3K27me3. **H.** The mRNA and protein levels of FPN were examined upon EZH2 knockdown in ZNF217-transfected LNCaP cells. **I-J.** The cell growth and intracellular iron level were assessed upon EZH2 knockdown in ZNF217-transfected LNCaP cells. Data depict the mean ± SD and representative of six independent experiments. Asterisk (^*^) indicates P < 0.05

Our previous study indicated that hypermethylation of FPN was the reason of FPN reduction in breast cancer [26]. In 2009, Banck et al reported that ZNF217 is a candidate organizer of repressive histone modifiers [11]. To this end, we hypnosis whether the above histone modification could also involve in ZNF217 mediating the suppression of FPN. As expected, depletion of ZNF217 reduced the ZNF217 and EZH2 occupancy of the FPN promoter (Figure 4A, P<0.05). In addition, ZNF217 knockdown also decreased the histone repressive markers (H3K9me3 and H3K27me3) and increased the histone active markers (H3K4me3) within FPN promoter (Figure 4A, P<0.05). In contrast, ZNF217 overexpression increased ZNF217 and EZH2 levels in the FPN promoter (Figure 4B, P<0.05). Besides, the H3K9me3 and H3K27me3 levels was increased and the H3K4me3 levels was decreased upon ZNF217 overexpression in LNCaP cells (Figure 4B, P<0.05). To determine whether overexpression of EZH2 leads to FPN attenuation, we assessed the mRNA expression of FPN by RT-PCR and protein levels by Western blot upon EZH2 knockdown or overexpression. As showed in Figure 4C-4F, downregulation of endogenous EZH2 inhibited the H3K27me3 levels and thus increased FPN1 mRNA and proteins levels, whereas EZH2 overexpression promotes H3K27me3 levels and thus suppressed the mRNA and protein levels of FPN (P<0.05). These data indicated that EZH2 has a critical role in regulating FPN expression. However, EZH2 knockdown or overexpression had no effect on the binding ability of ZNF217 to the promoter of FPN (Figure 4C, P<0.05). To investigate whether ZNF217 physically associated with EZH2 and H3K27me3, we performed Co-IP with specific antiZNF217, anti-EZH2 and H3K27me3 antibodies. As expected, endogenous ZNF217 co-immunoprecipitation with endogenous EZH2 and H3K27me3. The interaction of EZH2 and H3K27me3 with ZNF217 were further demonstrated by reverse endogenous Co-IP of EZH2 and H3K27me3 with ZNF217, supporting a physical EZH2-ZNF217 interaction *in vitro* (Figure 4G). Besides, we also examined the biological effect of the cooperation of ZNF217 and EZH2 in PCa by rescue experiments. As shown in Figure 4H, EZH2 knockdown largely restrained the suppression of FPN in LNCaP cells upon ZNF217 overexpression (P<0.05). Moreover, inhibition of EZH2 reversed the elevated cell growth and decreased cellular iron content in ZNF217-transfected LNCaP cells (Figure 4I–4J, P<0.05). These data together suggested that ZNF217 facilities the H3K27me3 levels of FPN promoter by cooperating with EZH2.

### MAZ transcriptional activates ZNF217 expression

**Figure 5:**
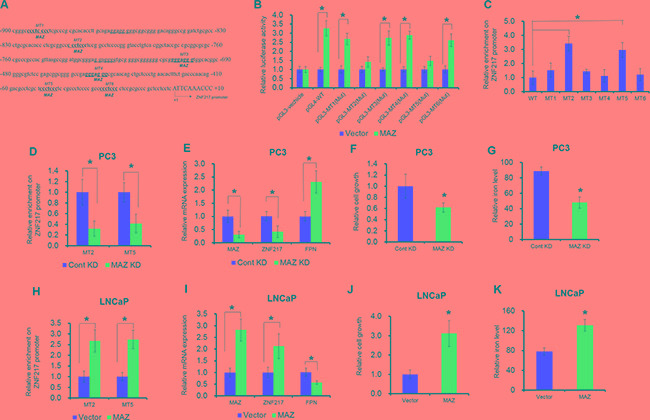
MAZ transcriptional activates ZNF217 expression **A.** Six putative MAZ binding sites (MT1-MT6) are underlined in the promoter of ZNF217 promoter region. +1 indicates the first nucleotide upstream of ghe transcription start site (TSS). **B.** Transcriptional activity of ZNF217 was determined by luciferase assay under MAZ overexpression. PC3 cells was transfected with pGL3-ZNF217 promoter (WT, MT1 mutation, MT2 mutation, MT3 mutation, MT4 mutation, MT5 mutation, MT6 mutation) when cells were transfected with MAZ plasmids. **C.** ChIP assay was performed to detect the six binding sites in ZNF217 promoter region using anti-MAZ antibody or normal IgG. **D, H.** The binding ability of MAZ on the promoter of ZNF217 was assessed upon MAZ knockdown in PC3 cells or overexpression in LNCaP cells. **E, I.** The mRNA expression of MAZ, ZNF217 and FPN were examined MAZ knockdown in PC3 cells or overexpression in LNCaP cells. **F, G, J, K.** The cell growth and intracellular iron content were determined in MAZ knockdown PC3 cells or MAZ-transfected LNCaP cells. Data depict the mean ± SD and representative of six independent experiments. Asterisk (^*^) indicates P < 0.05

We next endeavored to elucidate the upstream signaling responsible for the increased ZNF217 expression. To investigate the transcriptional machinery that controls ZNF217 expression, we screened the potential transcriptional factors by using an online software TFSEARCH and ALGGEN. As shown in Figure 5A, we identified six putative binding motifs of MAZ (CCCTCCC) within 1kp upstream from the transcription start site of ZNF217 gene. To assess the functional motifs on the promoter of ZNF217, we thus introduced site-directed mutation into each potential MAZ binding motif (MT1-MT6). Mutation on MT2 or MT5 abolished MAZ-mediated ZNF217 promoter transactivation in PC3 cells, whereas others motifs did not affect ZNF217 promoter activity. These results identified MT2 and MT5 as the functional motifs for the transcriptional activity of the ZNF217 gene in PC3 cells (Figure 5B, P<0.05). To verify whether MAZ physically binds to the ZNF217 promoter, we performed the ChIP assay with human MAZ antibody. As showed in Figure 5C, the ZNF217 promoter was indeed occupied in MT2 and MT5 by MAZ protein (P<0.05). In addition, ectopically expressed MAZ led to its accumulation at the ZNF217 promoter region, and this binding ability was undermined when MAZ expression was knockdown (Figure 5D, G, P<0.05). These results indicated the binding of MAZ to the ZNF217 promoter, and also implied their direct regulation of ZNF217. To validate the functional involvement of MAZ in modulating ZNF217 expression, we examined the ZNF217 expression by loss- and gain function of MAZ. Upon MAZ knockdown, the mRNA level of MAZ and ZNF217 were significantly decreased and the FPN expression was increased, relative to their scramble control (Figure 5E, P<0.05). Consequently, we also found both cell growth and the intracellular iron level was remarkably decreased (Figure 5F–5G, P<0.05). In contrast, ectopic expression of MAZ led to increase the binding ability on ZNF217 promoter and the mRNA level of MAZ in LNCaP cells, and elevated ZNF217 expression inhibited the FPN levels in MAZ-transfected LNCaP cells (Figure 5H–5I). Besides, we also found that overexpressed of MAZ promotes cell growth and the intracellular iron content in LNCaP cells (Figure 5J–5K, P<0.05). These results together demonstrated that MAZ transcriptional modulates ZNF217 expression.

### ZNF217 expression is upregulated in PCa samples

**Figure 6:**
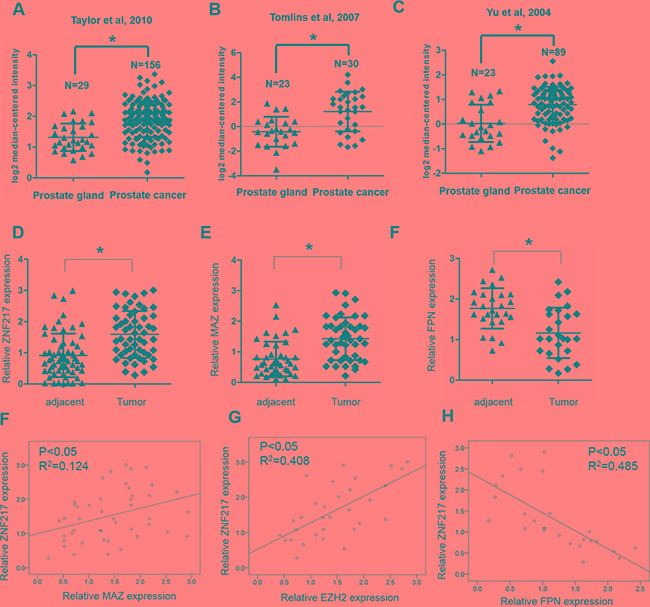
ZNF217 expression is upregulated in PCa **A, B, C.** Oncomine database showed that ZNF217 was significantly upregulated in PCa samples, compared with those of normal tissues. **D-F.** The mRNA expression of ZNF217, MAZ and FPN were assessed in our PCa samples and their adjacent tissues. **F, G.** Spearman correlation analysis revealed that ZNF217 expression was positively correlated with MAZ and EZH3 expression, and **H.** negatively correlated with FPN expression in PCa tissues. Data depict the mean ± SD and representative of six independent experiments. Asterisk (^*^) indicates P < 0.05

Finally, to further extend our observation to a clinical-pathological relevant context, we analyzed the expression of ZNF217 and its correlation with clinical behaviors of PCa patients. As shown in several microarray data from the Oncomine, the mRNA expression of ZNF217 was greatly increased compared to that in the healthy control (Figure 6A–6C, P<0.05). In the present study, we also identified the expression of ZNF217 was significantly upregulated in PCa samples when compared with those of adjacent tissues (Figure 6D, P<0.05). In addition, we also found that high ZNF217 expression was positively correlated with higher PSA levels, higher Gleason score, advanced tumor stage, lymph node metastasis and biochemical recurrence (Table 1, P<0.05). Moreover, elevated expression of MAZ and decreased expression of FPN were also found in PCa tissues compared to those of adjacent samples (Figure 6E–6F, P<0.05). Elevated ZNF217 levels was positively correlated with MAZ and EZH2 expression in PCa samples (Figure 6F–6G, P<0.05). Besides, we also found a negative association between ZNF217 and FPN levels in PCa samples (Figure 6H, P<0.05). Collectively, these findings implied abnormal ZNF217 expression plays a critical role in regulating iron-related PCa development.

**Table 1:** Relationship between ZNF217 and clinicopathologic variables

Feature		Total number (n)	ZNF217 expression		*P* value
			High (n)	Low (n)	
Age					
	≥70	33	16	17	0.79
	<70	22	12	10	
Serum PSA					**0.03**
	≥10	25	17	8	
	<10	30	11	19	
Gleason score					**<0.01**
	≥7	18	14	4	
	<7	37	14	23	
T stage					**0.01**
	≥T3	15	12	3	
	<T3	40	16	24	
Lymph node metastasis					**0.04**
	Presence	11	9	2	
	Absence	44	19	25	
Biochemical recurrence					**0.04**
	Presence	16	12	4	
	Absence	39	16	23	

## DISCUSSION

ZNF217 is a candidate oncogene located on chromosome 20q13.2, a region that is frequently amplified in many tumors, including those of the breast, colon, glioma, ovarian and prostate [8–10, 17, 29–31]. ZNF217 is a Kruppel-like finger protein that contains eight zinc fingers, suggesting it acts as a transcription factor [4, 5]. Recent studies showed that ZNF217 cooperates with several intracellular signaling networks to reprogram integrated circuits governing hall mark capabilities within cancer cells, including cellular immortalization, increased cellular proliferation, resistances to cell death and invasion activation. However, whether the aberrant expression of ZNF217 in regulating iron-related tumor progression remains largely unknown. In 2008, Thillainadesan et al demonstrated that p15(ink4b) act as a direct target of the ZNF217 complex. Downregulation of ZNF217 increased p15(ink4b) expression and coincided with increased in H3K4me3 and decrease in H3K19ac [32]. In addition, Sun and colleagues performed microarray analysis of gene expression in ovarian cancer with silencing of the ZNF217 gene. Silencing of ZNF217 resulted in significant down-regulated genes by at least 8-fold, including ALOX15, CD1D, FXYD3, GAS6, KRT4, LIN7B, MMP-24, PDZK1, PEX6, PRSS8, SLC2A9, STRN and WFDC2 [33]. In 2010, Krig et al reported that ZNF217 regulates the expression of ErbB3 receptor tyrosine kinase in breast cancer cells. They showed that ZNF217 recruitment to the ErbB3 promoter is CtBP1/2-independent and that ZNF217 and CtBP1/2 have opposite roles regulating ErbB3 expression [17]. eEF1A2 has been implicated in actin remodeling, invasion and migration, and silencing eEF1A2 inhibited the anchorage-independent growth mediated by ZNF217 in ovarian cells [34]. Besides, ZNF217 enrichment has been found in the promoter for Snail1 and Snail2 genes in human breast cancer, and the promoter of the E-cadherin gene is a direct target for ZNF217 [12, 14]. Our findings from this study established a critical role of ZNF217 in regulating iron-related cellular activities, including DNA replication and ATP production. Furthermore, we also uncovered that elevated ZNF217 significantly inhibited FPN expression to enhance the demand of iron in tumor cells.

Mammalian cells have a fine-tuned regulatory system to maintain systematic iron homeostasis [18–19]. However, aberrant iron levels could significantly trigger tumor initiation and progression through various mechanisms, such as excessive free radicals formation via iron-dependent Fenton reaction, elevated DNA synthesis and oxidative phosphorylation induced by iron. As the only known iron exporter in mammalian cells, FPN reduction lead to decreased iron export and increased intercellular iron that consequently aggravates the oncogenic effects of iron [20]. Previous studies indicated that FPN level is mainly controlled by hepcidin through protein internalization and degradation [23]. Besides, FPN could also be regulated by hypoxia inducible factor 2 (HIF2α), nuclear factor erythroid (Nrf2), metal regulatory transcriptioan factor 1 (MTF1), and myeloid zinc finger 1 (MZF1) at the transcriptional level in macrophages and tumor cells [26, 35–37]. Additionally, our recent study also verified the hypermethylation of FPN promoter was the reason of attenuated FPN expression in breast cancer cells [26]. However, whether histone modification also involved in regulating FPN expression remains largely unknown, and warranted close investigation. Recently, accumulating studies suggested that ZNF217 can be part of a transcriptional repressor complex of target genes [3, 4, 11, 15]. ZNF217 biochemically purifies with histone deacetylases HDAC1/2, histone demethylases LSD1, and Jarid1b, and histone methyltranferases G9a and EZH2, suggesting a range of regulatory functions in histone modifying complexes [11, 12]. EZH2 is a catalytic core component of PRC2 (polycomb repressive complex 2), which catalyzes the trimethylation of histone 3 at lysine 27 (H3K27me3). Overexpression of EZH2 have been proved to induce PCa progression [38]. In the present study, we found both EZH2 and ZNF217 could suppress the FPN expression in PCa cells. Consistent with previous study, we also identified a physical interaction between ZNF217 and EZH2 by CoIP assay [11]. ZNF217 knockdown significantly inhibited the binding ability of EZH2 to the FPN promoter, whereas deletion of EZH2 did affect the occupancy of ZNF217 to the promoter region of FPN. These results indicated that EZH2 is recruited to the FPN promoter, at least in part, through interaction with ZNF217. Our study together signified a novel function for ZNF217 in modulating FPN expression and eventually enhance tumorigenic effects of iron.

To further investigate the upstream signaling responsible for ZNF217's transcriptional regulation, we predicted its potential transcriptional factors using publicly available online algorithms. Then, we identified five putative binding motifs of MAZ within promoter region of ZNF217 gene. In 2011, Mao et al found knockdown of either HIF1α or HIF2α inhibited ZNF217 expression in glioblastoma. They also found HIF2α knockdown inhibited ZNF217 expression more efficiently in both normoxia and hypoxia than HIF1α knockdown [39]. Several miRNAs (miR-503, miR-200c, miR-24 and miR-203) targeting the 3’UTR of ZNF217 mRNA were found to regulate ZNF217 expression and functions [40–43]. Deregulated methylation status at the ZNF217 gene promoter has been observed in glioblastoma and breast cancer. LncRNA-ATB also regulates ZNF217 expression via miR-200c in keloid fibroblasts and breast cancer [44, 45]. In the current study, we found two functional binding sites of MAZ within the promoter of ZNF217 by ChIP assay and luciferase assay. MAZ, a transcriptional factor related to the development and progression of a variety of cancers, is highly expressed in PCa tissue and cell lines [46–48]. MAZ, a zinc finger transcription factor, is located on 16p11.2 and contains six C_2_H_2_-type zinc fingers at carboxyl terminus, a proline-rich region, and three alanine repeats. MAZ has been reported that negatively regulate miR-34a to promote breast cancer progression [49]. MAZ is also known to modulate PPARgamma1 expression to promote cell growth and inhibit apoptosis in breast cancer [50]. We here found elevated MAZ expression promoted ZNF217 levels at the transcriptional level, and consequently inhibited FPN concentration and iron-related cellular activities. Our current findings suggested the expression of ZNF217 was transcriptional activated by MAZ in prostate tumor cells.

**Figure 7:**
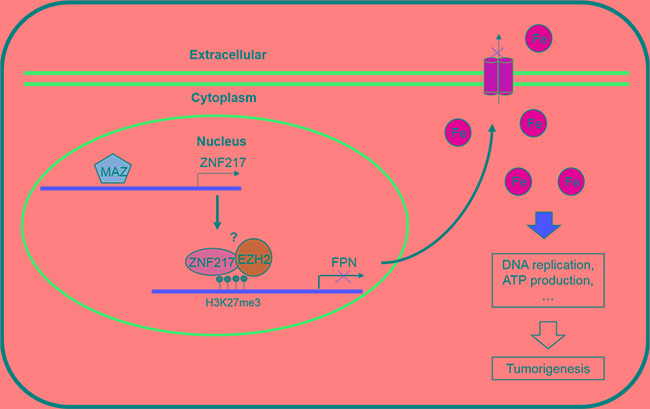
A scheme diagram deciphering the mechanism underlying ZNF217 exerts oncogenic role in prostate cancer by restraining ferroportin-conducted iron egress

In summary, this study overall deciphers a critical role of ZNF217 in inhibiting FPN expression and then restraining iron egress in prostate tumor cells (Figure 7). In addition, we also identified EZH2 as a component of ZNF217 complex to repress the FPN expression. Besides, MAZ have been proved to regulate ZF217 expression at transcriptional level. The results presented in this study established a novel role of ZNF217 in cellular iron metabolism, and also indicated that ZNF217 as a transcriptional repressor protein, at least in part, through limiting the tumorigenic effect of iron. Therefore, identification a novel oncogenic role of ZNF217 will be invaluable in gaining a better understanding of the role that ZNF217 plays in tumorigenesis.

## MATERIALS AND METHODS

### Patients and tissue samples

A total of 55 PCa patients and their adjacent samples were collected from the Second Hospital of Tianjin Medical University between July 2010 and July 2012. This study was approved by the Ethics Committee of Tianjin Medical University and informed consent was obtained from each patients. All the samples were stored at -80°C.

### Cell lines and reagents

The human PCa cell lines PC3 and LNCaP cells (ATCC) were maintained in RPMI-1640 culture median supplemented with 10% fetal bovine serum (Gibco, USA) and 100U/mL penicillin-streptoMAZin (Hyclone, USA). Cells were incubated at 37°C under humidified atmosphere with 5% CO_2_.

### Plasmid construction and transduction

Human ZNF217, EZH2 and MAZ cDNA were cloned into the pcDNA3.1 vector (Clonetech, USA). siRNA molecules of ZNF217, EZH2 and MAZ were purchased from GenePharma (China). Plasmid and siRNA transfection were performed using Lipfectamine^TM^ 2000 (Invitrogen, USA)

### Cell growth assays

Cell growth was determined by the Alarmar Blue assay following the manufacture's protocol (Sigma, USA). Briefly, cells were seeded in 96-well plates and starved in medium with 1% serum overnight. After 24 h treatment, resazurin was added into culture media at a final concentration of 10%, and then cells were cultured for additional 2 h. Thereafter, cells were washed with phosphate-buffer saline (PBS) three times and then fluorescence intensity was measured using a microplate reader. The excitation and emission wavelength were 530 and 590 nm, respectively.

### Cell cycle analysis

Cell cycle progression was determined following a standard protocol. Briefly, after the treatment, cells were washed with cold PBS, and then fixed in 70% ethanol overnight. Then, cells were re-suspended in 0.5 ml of PBS with 200 μg/ml RNase and 50 μg/ml propidium iodide and incubated for 30 min at 37°C. finally, 2.0 × 10^4^ cells were collected for flow cytometry analysis.

### DNA replication and ATP production assay

DNA replication was assessed through BrdU incorporation assay (Roche, USA). In short, cells were incubated with 10 μM BrdU overnight. Subsequently, cells were fixed by FixDenat for 30 min and then incubated with anti-BrdU-POD for 90 min at room temperature. After washed with PBS, cells were added 100 μl substrate solution. Finally, the immune complexes were detected according to the manufacturer's instructions. Intracellular ATP content was determined by an ATP assay kit according to the manufacture's protocol (Beyotime, China). Briefly, cells were washed twice with cold PBS and collected into lysis buffer (Solarbio, China). Relative cellular ATP levels were reflected by bioluminescent using a microplate reader.

### Liable iron pool (LIP) assay

The intracellular LIP levels were determined according to the standard calcein acetoxymethyl ester staining method (Sigma, USA). Briefly, cells were washed twice with cold PBS and treated with 0.5 μM calcein for 15 min at 37°C. After washed with cold PBS, cells were then divided into two parts. One part was treated with 100 μM desferoxamine for 1 h at 37°C and the other was left untreated. Finally, the intracellular fluorescence was measured by FACS analysis with excitation at 488nm and reading at 525nm. The LIP levels were calculated after deduction of the cellular fluorescence of deferoxamine-treated cells by that in untreated cells.

### RNA isolation, reverse transcription and quantitative real-time PCR (qRT-PCR) analysis

Total RNAs were extracted from cells using Trizol reagent according to the manufacturer's instructions (Invitrogen, USA). For the reverse transcription reaction, 2 μg total RNAs were used to synthesize first strand cDNA (TakaRa, China). Gene expression was determined using SYBR Green qPCR master mix (Roche, USA). GAPDH was used as an internal control. Primers were listed in Supplementary Tabel 1.

### Western blotting analysis

Culture cells were harvested and washed twice with cold PBS. Total proteins were extracted with RIPA lysis buffer (Solarbio, China) containing PMSF (Beyotime, China). Equal amounts of protein lysates were subjected to 10% SDS-PAGE. Antibodies were anti-GAPDH (1:1000, Proteintech, China), ferritin light chain antibody (1:500, Abcam, USA), FPN antibody (1:500, Sigma, USA), EZH2 and MAZ (1:1000, Cell signaling). The band intensities of Western blot were quantitated by Image J software, and normalized to those of loading control.

### Co-immunoprecipitation (Co-IP)

Co-IP was carried out with a kit from Pierce (USA). In short, cells were harvested and lysised in IP lysis buffer. The lysates were sonicated followed by centrifugation for 10 min at 1,2000 rpm at 4°C. The supernatants were pre-cleared by incubating them with protein A agarose for 1 h at 4°C. Antibodies were added to the supernatants for overnight incubation at 4°C, and then the beads were spun down for 10 min and re-suspended with IP lysis buffer. Finally, the supernatants were collected and assessed by western blot.

### Chromatin imunoprecipitation (ChIP) assay

Cells were harvested for ChIP by EZ-ChIP kit according to the manufacture's instruments (Millipore, USA). Briefly, cells were fixed with 1% formaldehyde for 10 min at room temperature. Then cells were harvested and washed three times with cold PBS. Fixed cells were incubated in lysis buffer for 5 min on ice. Then then samples were sonicated to shear DNA into fragments with 200-1000 base pairs. Protein G Agarose was added to antibody/chromatin complex and incubate overnight at 4°C. Immunoprecipitation DNA was purified using phenol-chloroform and ethanol precipitation, and then quantified using real-time qRT-PCR.

### Luciferase reporter assays

We constructed the ZNF217-Luc reporter within human ZNF217 promoter region. In detail, a 2k bp fragment of the gene for ZNF217 was inserted into the pGL3 vector. To analyze the significance of the MAZ binding sites (CCCTCCC) in the ZNF217 promoter, the sequence was mutated to CGGCTCC. The luciferase activity was measured by Dual-Luciferase Reporter Assay System (Promega, USA), as previously described [28].

### Tumor xengraft treatment model

All mouse experiments were performed under the protocols approved by the Institutional guidelines of the Second Hospital of Tianjin Medical University. Human PC3 and LNCaP cells (5.0 × 10^6^ cells/L) were subcutaneously injected into the right flanks of six-week old Balb/c nude mice. Tumor burdens were monitored by tumor volumes, and mice were sacrificed 6 weeks after injection.

### Statistical analysis

Two-tailed Student's t-test and one-way analysis of variance were used to analyze the experimental data. The SPSS Statistics 17.0 (SPSS, Inc., USA) was utilized to analyze the data. The data are represented as the mean ± SD. Statistical significance was determined with a *P* value < 0.05.

## SUPPLEMENTARY MATERIALS FIGURES AND TABLES


