# Supplementary material for: Elevated expression of ZNF217 promotes prostate cancer growth by restraining ferroportin-conducted iron egress
Source: Oncotarget. 2016 Oct 19;7(51):84893–906. doi: 10.18632/oncotarget.12753 (PMC5356707; doi:10.18632/oncotarget.12753)
Supplement: Supplementary file 1 [file oncotarget-07-84893-s001.pdf]

Elevated expression of ZNF217 promotes prostate cancer growth by restraining ferroportin-conducted iron egress

SUPPLEMENTARY TABLE

Supplementary Table 1: Primer list

qRT-PCR		
GAPDH	forward	GGAGCGAGATCCCTCCAAAAT
	reverse	GGCTGTTGTCATACTTCTCATGG
ZNF217	forward	GTTGTTCCATTCCGAGCTACA
	reverse	GGTAGGCCGGTGTTGCATTA
FPN	forward	CTACTTGGGGAGATCGGATGT
	reverse	CTGGGCCACTTTAAGTCTAGC
EZH2	forward	AATCAGAGTACATGCGACTGAGA
	reverse	GCTGTATCCTTCGCTGTTTCC
MAZ	forward	ACCACCTGAACCGACACAAG
	reverse	AAGCTGCCTCACATTTCTCAC
ChIP-qPCR		
ZNF217	forward	GATAGCAGCCGCAGAAGA
	reverse	TCACAGCAGAGCCACATT
MT1	forward	CTTCTAACCCGCGAGCAA
	reverse	CACCCCGAATTACAAACAAA
MT2	forward	CTTCTAACCCGCGAGCAACGA
	reverse	AGGCGCAGATCCGGCCCTGT
MT3	forward	TGACAGCCTCCCGCCATCC
	reverse	CTACCGCCTCCTCCCCA
MT4	forward	AGGGAGGGCGCAACAGCT
	reverse	CACTCCCAGTTCTCGCTCGG
MT5	forward	TAAGCCGGATGGCGGGAGGA
	reverse	GGAGGCGCGGAGTTTCT
MT6	forward	CCGCCCTCCCCGCCCCTC
	reverse	CCGGGTTTAATGAGGAGGA
